# Novel monoclonal antibodies to normal and pathologically altered human TDP-43 proteins

**DOI:** 10.1186/2051-5960-2-33

**Published:** 2014-03-31

**Authors:** Linda K Kwong, David J Irwin, Adam K Walker, Yan Xu, Dawn M Riddle, John Q Trojanowski, Virginia M Y Lee

**Affiliations:** 1 Center for Neurodegenerative Disease Research & Institute on Aging, Department of Pathology and Laboratory Medicine, Perelman School of Medicine, University of Pennsylvania, Philadelphia, PA 19104-4283, USA; 2 Penn Frontotemporal Degeneration Center, Department of Neurology Perelman School of Medicine, University of Pennsylvania, Philadelphia, PA 19104-6021, USA

**Keywords:** TDP-43, Monoclonal antibody characterization, FTLD-TDP, ALS, Biomarker

## Abstract

The RNA/DNA-binding protein, TDP-43, is the key component of ubiquitinated inclusions characteristic of amyotrophic lateral sclerosis (ALS) and the majority of frontotemporal lobar degeneration (FTLD-TDP) referred to collectively as TDP-43 proteinopathies. To further elucidate mechanisms of pathological TDP-43 processing and identify TDP-43 epitopes that could be useful as potential biomarkers of TDP-43 proteinopathies, we developed a panel of novel monoclonal antibodies (MAbs) directed at regions extending across the length of TDP-43. Here, we confirm previous observations that there is no or minimal accumulation of TDP-43 N-terminal domains in neocortical inclusions in human TDP-43 proteinopathy tissues and we identify a subset of these MAbs that are specific for human versus mouse TDP-43. Notably, one of these MAbs recognized an epitope that preferentially detected pathological TDP-43 inclusions with negligible reactivity for normal nuclear TDP-43 resembling anti-phospho-TDP-43 specific antibodies that only bind pathological TDP-43. Hence, we infer that this new MAb recognizes a phosphorylation independent but disease-specific pathologic conformation in abnormal TDP-43. These data suggest that the novel MAbs reported here will be useful for patient-oriented research as well as for studies of animal and cell-based models of TDP-43 proteinopathies including ALS and FTLD-TDP.

## Introduction

Since the landmark discovery of the RNA/DNA binding protein, TDP-43, as the main constituent of ubiquitin-positive, tau/synuclein-negative inclusions in amyotrophic lateral sclerosis (ALS) and frontotemporal lobar degeneration (FTLD) referred to as FTLD-TDP [1], there have been numerous advances in our understanding of these seemingly disparate clinical and neuropathological syndromes (reviewed in [2]). Indeed, ALS and FTLD-TDP are now considered to be different manifestations along a continuum or spectrum of TDP-43 proteinopathies [3]. Further, hierarchical non-random progression of TDP-43 pathology in ALS [4] and FTLD-TDP [5] suggest potential neuron-to-neuron spread of TDP-43 as a major driving force in the progression of the neurodegenerative process. Indeed, cell culture experiments suggest the possibility of transmission of pathological TDP-43 between cells [6] similar to those reported in animal and cellular models of tauopathies [7,8] and Parkinson’s disease [9-12]. Accordingly, strategies to prevent or slow the transmission of pathogenic forms of TDP-43 in affected patients with passive immune therapy using disease specific monoclonal antibodies (MAbs) to pathological TDP-43 could emerge as a major therapeutic approach for treatment of FTLD-TDP and ALS. To that end and for future research on animal- and cell-based models of TDP-43 proteinopathies, well-characterized MAbs to normal and pathological epitopes in TDP-43 are urgently needed. Here we describe the generation and characterization of novel MAbs that detect normal and disease-specific epitopes spanning the length of TDP-43.

## Materials and methods

### Recombinant TDP-43 production

Human recombinant TDP-43 (rTDP-43) proteins including full length TDP-43 (FL-rTDP-43), i.e. amino acids (aa) 1–414, N-terminally truncated TDP-43 (Nt-rTDP-43), aa 1–261, and C-terminally truncated TDP-43 (Ct-rTDP-43), aa 182–414 were expressed in BL21 (DE3) *E. coli* cells using the pCOLD vector system (Takara Bio Inc., Japan). TDP-43 proteins were extracted from *E. coli* 16 h after IPTG induction, and the vast majority of the rTDP-43 proteins were present in inclusion bodies. The bacterial pellet was sequentially extracted with PBS and 1% Triton X-100 to remove soluble bacterial proteins. For immunization, the Triton X-100 insoluble rTDP-43 was solublized with 1% Sarkosyl buffer or 4 M urea. Sarkosyl was removed by exhaustive dialysis before use. For epitope mapping, the Triton X-100 insoluble rTDP-43 was solublized directly in Laemmli sample buffer, and immunoblot (IB) analysis was done as described previously [13,14].

### MAb generation and screening

Murine MAbs were raised against human rTDP-43 proteins using similar methods described previously [15-18]. Mice were immunized with FL-rTDP-43 or Nt-rTDP-43. Briefly, rTDP-43 proteins were emulsified with Freund’s adjuvant and injected subcutaneously into BALB/c mice. The mice were boosted 3 additional times at 2 week intervals. Three days prior to harvesting spleens for fusion to generate MAbs, the mice received intraperitoneal injection of rTDP-43 without adjuvant. Splenic lymphocytes were fused to Sp2 mouse myeloma cells using the polyethylene glycol method to produce mouse hybridomas as described [15]. Animal care and all procedures performed here were conducted in accordance with the NIH Guide for the Care and Use of Experimental Animals and approved by the University of Pennsylvania Institutional Animal Care and Use Committee. Hybridoma supernatants were initially screened for the ability to recognize human FL-rTDP-43 by indirect enzyme-linked immunosorbant assay (ELISA), and positive clones were further evaluated by IB using FL-rTDP-43 and by immunohistochemistry (IHC) on formalin and ethanol fixed paraffin-embedded sections of FTLD-TDP cortex (see below). Cells from positive hybridomas were expanded and re-screened as above.

### Epitope mapping and characterization of MAbs

The TDP-43 domains harboring the epitopes recognized by these new MAbs were first estimated using FL-rTDP-43, Nt-rTDP-43 and Ct-rTDP-43 in IB studies similar to our characterization of MAbs we previously generated to tau and alpha-synuclein [18,19]. After probing with the MAbs, the bound antibodies were detected with horseradish peroxidase-conjugated goat anti-mouse IgG (Jackson ImmunoResearch, West Grove, PA). The blots were visualized using the chemiluminescent system as described previously [1]. For more refined mapping of the TDP-43 protein domains harboring the epitopes detected by these MAbs, indirect ELISAs were performed using peptides corresponding to different regions spanning the length of TDP-43: aa 6–24, 40–54, 110–147, 175–187, 189–195, 203–220, 235–243, 251–263, 287–322, 341–352, and 394–414. Cross-reactivity to mouse TDP-43 was evaluated by IB using RIPA solubilized human and mouse cell lysates [20] and by IHC using paraffin-embedded mouse and human brain sections (see below). To determine human specificity, the immunoreactivity of each MAb to human versus mouse TDP-43 was compared as described by Giasson et al. [19]. Similarly, immunocytochemistry (ICC) was performed using QBI293 and Neuro2a cells to verify cross-reactivity [20]. Human cortical urea extracts from a normal control and a FTLD-TDP case were used to determine if the MAbs recognized the pathological signature of TDP-43 by IB [1]. Isotypes of the MAbs were determined using a commercial rapid ELISA mouse antibody kit (Thermo Fisher Scientific, Rockford, IL, USA).

### Sandwich ELISA

The 384-well format sandwich ELISA method used to evaluate the MAbs was similar to those described previously for a 96-well format, except the sample volumes used were reduced to 30 μL/well [21]. Briefly, plates were coated with 5 μg/mL of capture antibody (rabbit polyclonal Ct or Nt TDP-43 (pAb) or MAbs) and incubated overnight at 4°C. Plates were blocked for a minimum of 3 days at 4°C with Block-Ace (AbD Serotec, Raleigh, NC). After the blocking solution was removed, 0, 1 or 10 ng/mL of FL-rTDP-43 were added to wells and incubated for 16 h at 4°C. Plates were subsequently washed and incubated with 0.2, 1 or 10 μg/mL, detection antibody (MAbs for the rabbit pAb coated plates and rabbit pAb for MAb coated plates) for 16 h at 4°C. The plates were then washed and incubated with HRP-conjugated reporting antibodies (goat anti-mouse or anti-rabbit, respectively), for 1 h at 25°C (Santa Cruz Biotechnology). A tetramethyl benzidine peroxidase substrate system was used for the color development step (Thermo Fisher Scientific, Rockford, IL, USA). A serial diluted rTDP-43 standard curve, 10 ng/mL to 1 pg/mL, was used to estimate the level of detergent soluble TDP-43 in brain tissue or cell extracts. Samples were done in quadruplicates. Detection limit is defined as average optical density of reagent background (no rTDP-43 added) plus 4 times the standard deviation.

### IHC studies

Human CNS tissues from the superior temporal cortex (FTLD-TDP subtypes A-D) and spinal cord (ALS) (one patient each) were examined to evaluate the reactivity of TDP-43 MAbs in pathological aggregations and neuronal nuclei (for demographic details please see Additional file 1: Table S1). Neuropathological examination of human cases was performed as described previously [22] using current neuropathological criteria for the diagnosis of FTLD-TDP and ALS [23,24]. The brain and spinal cord tissues were obtained at autopsy and processed following standard established protocol [22]. Autopsy and required informed consent were performed in accordance with the University of Pennsylvania Institutional Review Board. Paraffin-embedded human CNS tissue sections were deparaffinized in xylenes and rehydrated in a descending series of ethanol. Endogenous peroxidases were blocked with 5% hydrogen peroxide in methanol, and sections were microwaved in 1% antigen unmasking solution (Vector Laboratories, Burlingame, CA) at 99°C for 15 min, blocked in 2% fetal bovine serum in 0.1 M Tris buffer and incubated with TDP-43 MAbs in ascites or as purified IgG. Affinity-purification of IgG was done using the HiTrap Protein G column according to manufacturer instructions (GE Healthcare, Piscataway, NJ). MAbs were incubated overnight at 4°C at concentrations of 1:1 K-1:100 K (ascites) or 0.03-1.0 μg/ml (purified IgG). Reactivity of novel TDP-43 MAbs was compared with a previously characterized disease-specific MAb to TDP-43 phosphorylated at serine 409 and 410 (MAb p409/410) [25] at concentrations of 1:250–1:500.

IHC of mouse CNS tissues was performed in the same manner described above, and in addition, we used a mouse on mouse (MOM) immunodetection kit (Vector Laboratories) to minimize endogenous mouse IgG background. For all sections, labeling was detected using species-specific biotinylated secondary antibodies incubated for 1 h and the Vectastain ABC kit followed by the ImmPACT*™* DAB Substrate kit (Vector Laboratories). Slides were coverslipped with CytoSeal 60 mounting medium (Thermo Scientific).

The degree of non-pathological nuclear TDP-43 and pathological TDP-43 deposits in human lower motor neurons (LMN), white-matter glial cell inclusions (GCI), neuronal cytoplasmic inclusions (NCI), dystrophic neurites (DN) and neuronal nuclear inclusions (NII) were reviewed by a trained examiner (DJI) and rated on a semi-quantitative scale (0 = none, r = rare, + = few, ++ = moderate, +++ = many) for each MAb. Digital images of IHC results were obtained using an Olympus BX 51 microscope equipped with a bright-field and fluorescence light source with a DP-71 digital camera (Olympus; Center Valley, PA) and DP manager software (Olympus).

## Results

### MAb generation and epitope mapping

**Figure 1:**
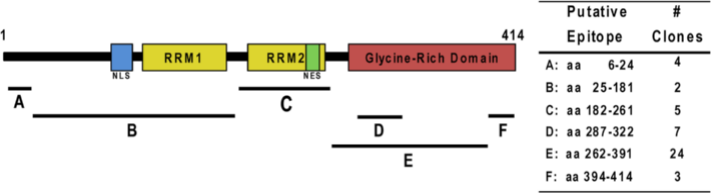
**Schematic representation of TDP-43 domains containing epitopes recognized by the MABs reported here.** The TDP-43 domains harboring the epitopes recognized by 45 newly generated MAbs were mapped to regions along the entire length of TDP-43 **(A-F)** by immunoblot and indirect peptide ELISA and the putative amino acid stretches included in these epitopes is depicted in the table to the right of the schematic. RRM: RNA-recognition motif; NLS: bipartite nuclear localization signal; NES: nuclear export signal.

To gain further insights into the molecular and neuropathological roles of TDP-43 in neurodegeneration, we generated new MAbs for future studies of TDP-proteinopathies. Approximately 4500 potential MAb clones from three hybridoma fusion procedures were screened by FL-rTDP-43 indirect ELISA of which 168 clones were positive on this initial screen and all were evaluated further in IB and IHC studies. Only 45 clones continued to show immunoreactivity toward TDP-43 in the secondary screens and all of these MAbs were studied further to determine the epitopes and their antibody isotype as described [18,19]. Using FL-rTDP-43, Nt-rTDP-43, and Ct-rTDP-43 for IB analyses, the epitope region was estimated to be within aa 1–181 for 6 MAbs, within aa 182–261 for 5 MAbs, and within aa 262–414 for 34 MAbs (data not shown and Table 1). Indirect ELISA using a panel of TDP-43 peptides as antigens further mapped some of the epitopes to smaller regions of TDP-43. Four of the MAbs recognized the aa 6–24 peptide, 7 recognized the aa 289–322 peptide and 3 recognized the extreme C-terminus peptide (aa 394–414) (Figure 1). After additional cell passages and/or subcloning procedures, 18 MAbs were selected for further characterization based on the hybridomas’ apparent clonality and stability. These MAbs were shown to have their putative epitopes mapped to the regions in TDP-43 shown in Table 1. Next, these MAbs were evaluated for their effective use in several assays including: immunoprecipitation and IB of human cell lysates, IHC, ICC, and sandwich ELISA. Most of the MAbs were found to have efficacy in all assays tested, except for MAb 211 (Table 1) which failed as a detection antibody by ELISA even at a 20 fold higher concentration than that used for other MAbs (data not shown).

**Table 1:** Summary of the characterization of New TDP-43 MAbs

**MAb**	**Isotype**	**Epitope (aa)**	**IP**	**IB**	**IHC**	**ICC**	**ELISA capture**	**ELISA detect**	**Human preference**
211	IgG_1_	6-24	-	+	+	+	+	-	-
5031	IgG_2a_	25-181	+	+	+	+	+	+	-
138	IgG_2a_	182-261	+	+	+	+	+	+	+
5060	IgG_2a_	182-261	ND	+	+	+	+	+	-
5117	IgG_2a_	287-322	ND	+	+	+	+	+	-
5156	IgG_2a_	287-322	+	+	+	+	+	+	-
205	IgG_2a_	262-391	+	+	+	+	+	+	+
241	IgG_1_	262-391	+	+	+	+	+	+	+
5028	IgG_1_	262-391	+	+	+	+	+	+	-
5032	IgG_1_	262-391	+	+	+	+	+	+	+
5053	IgG_1_	262-391	ND	+	+	-	+	+	-
5056	IgG_2a_	261-391	+	+	+	+	+	+	-
5089	IgG_2b_	261-391	+	+	+	+	+	+	-
5095	IgG_1_	261-391	+	+	+	+	+	+	+
5104	IgG_1_	261-391	+	+	+	+	+	+	+
5123	IgG_1_	261-391	+	+	+	+	+	+	+
5101	IgG_1_	261-391	+	+	+	+	+	+	-
5195	IgG_2a_	394-414	+	+	+	+	+	+	-

This table summarizes the properties the new MAbs described here. New abbreviation: ND = not determined.

**Figure 2:**
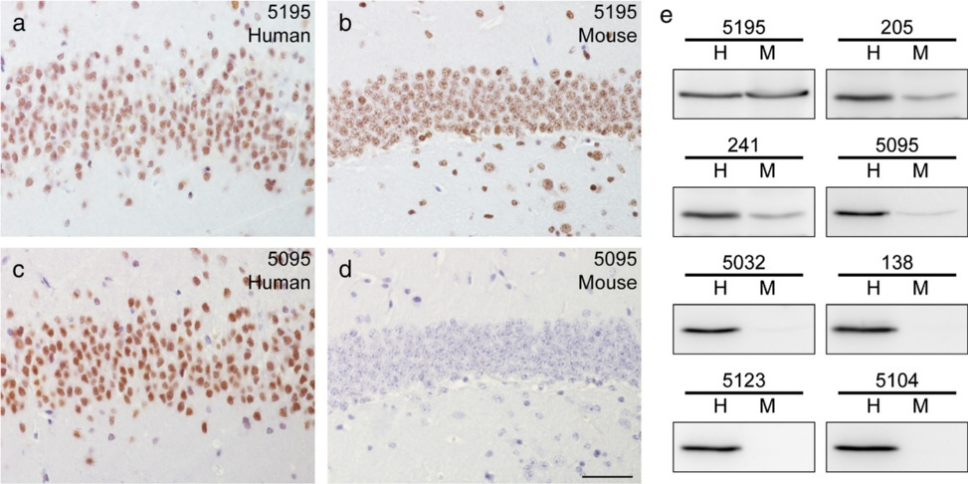
**Representative IHC staining patterns and immunoblot profiles of selected MAbs that recognize human- or human- and mouse-TDP-43.** MAb 5195 detects normal nuclear TDP-43 in **(a)** human and **(b)** mouse hippocampus dentate gyrus. In contrast, MAb 5095 detects **(c)** human but not **(d)** mouse TDP-43, which is representative of 7 MAbs that preferentially recognize human TDP-43. Scale bar = 50 μm. **(e)** All the MAbs here show strong immunoreactivity with a 43 kDa band from human cell lysates, but 7 MAbs preferentially recognize human TDP-43 with little or no detection of mouse TDP-43. MAb clone numbers are shown above each immunoblot, and MAb 5195 is representative of 8 MAbs that detect human and mouse TDP-43 with equal affinity. H – human, M – mouse.

To determine whether or not the selected 18 MAbs detected both human and mouse TDP-43, IHC was performed using normal human and non-transgenic mouse brain sections. All 18 MAbs detected nuclear TDP-43 in sections of human hippocampus (Figure 2a,c), but 7 of these MAbs did not detect mouse TDP-43 by IHC (compare Figure 2b to Figure 2d). To determine if these 7 apparently human specific MAbs detect mouse TDP-43 in other applications, we compared their immunoreactivity toward human versus mouse TDP-43 from cultured cells on IB. Only 3 of these 7 MAbs (138, 5123, and 5104) did not detect the mouse protein. The remaining 4 MAbs, including MAbs 5032 and 5095, recognized mouse TDP-43, albeit with lower efficiency than the human protein (Figure 2e). Thus, we identified 11 MAbs that detected both mouse and human TDP-43 with equal affinity, 4 that preferentially recognized human versus mouse TDP-43 but were not completely human specific, and 3 that detected human but not mouse TDP-43 in the assays used here.

### ELISA studies

As of now, there is no reliable method to quantify TDP-43 proteins in biofluids such as plasma or cerebrospinal fluid (CSF) samples which would be useful as a biomarker assay. In order to determine if the MAbs generated in this study might be useful for developing an assay to detect TDP-43 in CSF or plasma, we evaluated them in a sandwich ELISA using previously developed Nt- or Ct-TDP-43 pAbs [17] as capture or detection antibody. As mentioned above and shown in Table 1, 17 MAbs were capable of detecting and capturing FL-rTDP-43 by ELISA. This suggested that one or more of these MAbs may be advanced for the development of a sensitive ELISA to quantify TDP-43, especially in biofluids. This is exemplified by studies using MAb 205 for capture and the Ct-pAb as the detection antibody in an ELISA that showed a lower limit of detection for human rTDP-43 of ~25 pg/mL (see Additional file 1: Figure S1). Using this sensitive ELISA, we were able to quantify the amount of detergent soluble TDP-43 in QBI293 cell lysates and in human FTLD-TDP cortical grey matter lysates, ~30 ng/mg protein and ~850 ng/g grey, respectively.

### Immunoreactivity in human tissue

**Figure 3:**
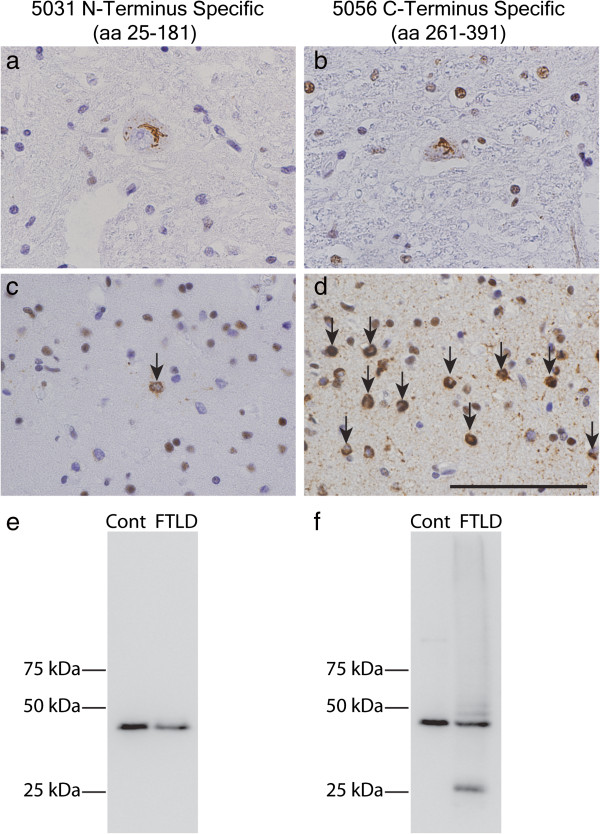
**IHC and IB properties of new N- and C-terminal specific anti-TDP-43 MAbs.** N-terminal specific MAb 5031 reacts with spinal cord ALS TDP-43 pathology **(a)** with minimal detection of FTLD cortical TDP-43 pathology (arrow, **c**), while MAb 5056 shows robust reactivity for both spinal cord **(b)** and cortical TDP-43 inclusions (arrows, **d**). Consistent with previous studies [13], immunoblot analysis shows that MAb 5031 detects full-length but not the CTF of TDP-43 **(e)**, while C-terminal specific MAb 5056 recognizes both **(f)**. Scale bar = 100 μm.

TDP-43 MAbs were screened for reactivity in human CNS tissues using IHC in the spinal cord of ALS and superior temporal cortex of morphological subtypes of TDP-43 (Type A-D) [24]. Semi-quantitative analyses for each type of morphologic inclusion (i.e. LMN, NCI, DN, NII, and GCI) in ALS and FTLD-TDP are summarized in Table 2. MAbs 211 and 5031, which are specific for the N-terminus of TDP-43 (i.e. regions A and B as illustrated in Figure 1) showed robust reactivity for normal nuclear TDP-43, but 5031 detected more pathology in the ALS spinal cord and FTLD-TDP temporal cortex than 211 (Figure 3a,c and data not shown). Indeed, the amount of pathologic TDP-43 in cortical sections detected by 5031 was modest, while 211 did not detect significant amounts of neocortical TDP-43 deposits in the FTLD-TDP cases. Thus, the N-terminal specific MAbs preferentially detected TDP-43 pathology mainly in spinal cord motor neurons, which contains a predominance of full-length TDP-43 [13]. In contrast, MAbs specific for the RNA recognition motif 2 (RRM2), i.e. Region C in Figure 1 or the C-terminal glycine rich domain (i.e. Regions D-F) were immunoreactive to all forms of neocortical pathological TDP-43 inclusions seen in FTLD-TDP subtypes, as well as LMN inclusions in ALS from a moderate to high degree for most inclusion types (Figure 3b,d). The minimal limited immunoreactivity of MAbs specific for N-terminal regions in neocortical TDP-43 inclusions of FTLD-TDP cases was confirmed using IB analyses of human CNS urea extract of FTLD-TDP brains which showed that a C-terminal fragment (CTF) of TDP-43 was detected by MAbs specific for regions in the C-terminus (Figure 3f), but not the N-terminus specific MAb 5031 (Figure 3e). Moreover, MAb 5031 also did not detect the pathological hyperphosphorylated 45 kD band or the high molecular weight (MW) smears which were detected MAb 5056, consistent with previous findings [13].

**Table 2:** IHC properties of new TDP-43 MAbs

**Region/antibody**	**Spinal cord**			**Temporal cortex**				
	**Nuclei**	**LMN**	**GCI**	**Nuclei**	**NCI**	**DN**	**NII**	**GCI**
A (aa 6–24)								
211	+++	+	+	+++	0	0	0	0
B (aa 25–181)								
5031	++	+++	+	++	r	+	+	r
C (aa 182–261)								
138	0	+++	+	0	+++	++	+	+
5060	+++	+++	+	++	+	+	++	r
D (aa 287–322)								
5117	++	++	r	+++	+++	++	r	++
5156	+++	+++	r	+++	+++	+++	+	++
E (aa 262–391)								
205	+++	+++	r	++	+++	++	+	+
241	++	+++	+	++	+++	++	+	+
5053	+++	+++	+	+++	+++	+++	++	+
5056	++	++	r	+++	+++	+++	++	+
5095	+++	++	r	+++	++	++	++	+
F (aa 392–414)								
5195	+	+++	r	+++	++	++	++	+

This table summarizes the IHC characteristics of the new MAbs described here. They are identified by the numbers in the first column under A-F. The aa numbers in parenthesis indicates the different protein domains within which the epitopes reside that are recognized by these new MAbs. New abbreviations: Nuclei = Normal endogenous TDP-43 staining; LMN = lower motor neuron cytoplasmic inclusions, r = rare.

**Figure 4:**
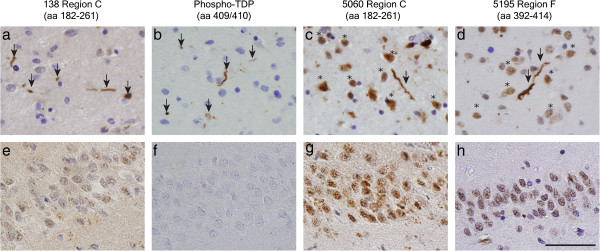
**Preferential recognition of pathological TDP-43 by MAb 138.** MAb 138 robustly detects pathological TDP-43 inclusions (arrows) with no or very minimal nuclear staining in FTLD temporal cortex **(a)** and little recognition of normal nuclear TDP-43 in human control dentate gyrus **(e)**, similar to previously described antibodies specific for phospho TDP-43 **(b, f)**. In contrast MAb 5060, the epitope for which lies within aa 182–261, and MAb 5195 which detects an epitope contained within aa 394–414, recognize both pathological TDP-43 inclusions (arrows) and normal nuclear TDP-43 (asterisks) in FTLD temporal cortex **(c,d)** and control dentate gyrus **(g,h)**. Scale bar = 50 μm.

The reactivity to neocortical TDP-43 pathology by MAbs specific for C-terminal TDP-43 epitopes was accompanied by a moderate to high reactivity for normal nuclear TDP-43 by all MAbs except MAb 138. MAb 138, which is specific for region C in the RRM2, displayed high reactivity to pathological TDP-43 deposits similar to other TDP-43 MAbs specific for Regions C-F, in the absence of significant normal nuclear TDP-43 staining (Figure 4). This preferential detection of pathological TDP-43 inclusions with minimal or no normal nuclear TDP-43 staining by IHC is similar to the staining pattern seen with pathology-specific phospho-TDP-43 specific MAbs such as the p409/410 MAb mentioned above. There was no regional predilection for this pattern, as it was evident in both the neocortex and spinal cord of ALS and FTLD-TDP patients. To further test this observation we analyzed the hippocampal dentate gyrus from a non-neurodegenerative control case with MAb138 under a range of dilutions to establish if the negligible degree of normal nuclear TDP staining was reproducible in a control case. As shown in Online Resource 1 (Additional file 1: Figure S2), the pattern of immunoreactivity observed with MAb 138 did not appear to reflect antibody dilution or affinity. Finally, there were no clear differences between the ability of the different MAbs to detect the different morphological types of TDP-43 inclusions (i.e. DN, NCI, NII, GCI and granular cytoplasmic TDP deposits), FTLD-TDP subtypes A-D as classified by MacKenzie et al. [24] or clear effects of fixative method or post-mortem interval in this screening survey.

## Discussion

Here we describe the generation and characterization of several novel TDP-43 specific MAbs that recognize epitopes distributed over the entire length of TDP-43 (Figure 1, Table 1) most of which are useful for immunoprecipitation and IB methods, as well as for use as capture or detection MAbs in an ELISA assay in addition to being effective in ICC and IHC studies. Notably, using MAb 205 in a sandwich ELISA as the capture antibody, we were able to detect human rTDP-43 at a lower limit of 25 pg/ml (Additional file 1: Figure S1). Moreover, we also identified a subset of 7 MAbs that preferentially detect human TDP-43 and not mouse TDP-43 while we showed that 11 of these new MAbs detect both human and mouse TDP-43 with equal robustness. Further, using these new MAbs, we confirm previous results [13] showing a predominance of CTF in neocortical TDP-43 inclusions of FTLD-TDP brains and full-length TDP-43 in the spinal cord of ALS spinal cord (Figure 3). Remarkably, we identified a unique MAb (138) that binds to an epitope in the RRM2 region that shows a preferential reactivity for pathological TDP-43 inclusions and minimal or no recognition of normal nuclear TDP-43, similar to disease specific phospho-TDP-43 specific MAbs such as the p409/410 MAb (Figure 4).

Since mouse TDP-43 shares 96% identity with the human protein, it is not uncommon for antibodies made against human TDP-43 to react with the mouse protein. Surprisingly, in this study we identified 7 MAbs that preferentially recognize human TDP-43, 6 of which bind to an epitope in regions downstream of aa 261. These MAbs are all human specific on IHC, but have varying immunoreactivity on IB to mouse TDP-43, from none detected to ~30% cross-reactivity. The varying reactivity of these novel MAbs to mouse TDP-43 most likely reflects underlying differences in aa sequence between human and mouse TDP-43. Thus, these novel and relatively human-specific TDP-43 MAbs will be very useful for studies of transgenic animal models of TDP-43 proteinopathies, including differentiating mouse versus human TDP-43 CTF that accumulate in pathologic inclusions.

Our IHC studies in human ALS and FTLD-TDP tissues with these novel MAbs suggests they may provide insights into the pathophysiology of TDP-43 misfolding and aggregation in human disease. First, our results reinforce previous findings [13] of a predominance of CTF in neocortical inclusions in FTLD-TDP, confirmed by minimal reactivity of our novel N-terminus specific MAbs (i.e. regions A-B) with brain TDP-43 pathology. Interestingly, MAb 211, which is specific for the extreme N-terminus (Region A), did not detect significant neocortical pathology while MAb 5031 (Region B) detected rare to low levels of neocortical pathology (Figure 3). Furthermore, MAb 5031 did not appear to favor the detection of a particular cell type (i.e. oligodendrocyte, astrocyte, neurons) or cellular inclusions type (i.e. NCI, DN, etc.) suggesting the lack of alternative processing of pathological TDP-43 within different cell types in the neocortex of FTLD-TDP brains. However, the cause of preferential accumulation of full-length TDP-43 into LMN neuron and glial inclusions in the spinal cord of ALS remains unclear.

IB analysis of human brain urea extracts also showed that the Nt MAbs had limited immunoreactivity for the pathological biochemical features of FTLD-TDP. MAb 5031 detected the 43 kD band with similar sensitivity as the Ct MAb 5056; however, MAb 5031 failed to detect the Ct fragment, as well as the 45 kD band and the high MW smear. This finding is consistent with our previously developed Nt-pAb, which had much less immunoreactivity toward the pathological TDP-43 on IB when compared to the Ct-pAb [13]. It is possible that conformational changes in pathological TDP-43 render the Nt epitope inaccessible to the antibody by immunoblotting. However, we cannot rule out the possibility that the majority of protein species in the 45 kD band and the high MW smear might not be full length TDP-43.

MAbs specific for presumed sequences from aa 182 and beyond towards the C-terminus of TDP-43 (i.e. Regions C-F) showed robust reactivity for neocortical TDP-43 pathology across FTLD-TDP subtypes and ALS spinal cord inclusions. These MAbs directed against epitopes in the mid- and C-terminus of TDP-43 detected a moderate to abundant normal nuclear TDP-43 with the exception of MAb 138 (Region C) (Figure 4). MAb 138 has a higher affinity for pathological TDP-43 similar to the pathology-specific MAb p409/410. MAb 5060 was also mapped to Region C, but did not share this unique IHC characteristic with MAb 138. It is unlikely that the 2 MAbs have identical epitope within Region C, which spans 79 aa. Thus, further epitope mapping is needed to determine their specific epitopes within this region and clarify the discordant nature of the IHC reactivity between these MAbs.

There are several possible explanations for the unique IHC staining by MAb 138. First, there is a disease-specific pathological modification to one or more aa within the epitope recognized by MAb 138, but this seems unlikely as all these novel MAbs were generated using un-modified human rTDP-43. Second, a recent report suggests that the RRM2 region can undergo self-association when interacting with nucleic acids and this could alter the conformation of TDP-43 or the accessibility of the epitope recognized by MAb 138 [26]. Finally, it is possible that under physiological conditions the MAb 138 specific epitope may be buried or bound to nucleic acids and not be accessible to the antibody when present normally in the nucleus while becoming more accessible in TDP-43 inclusions. Of note, MAb 138 does detect the normal nuclear TDP-43 in QBI293 cells by ICC (Table 2), albeit to a much less degree or intensity than the other MAbs reported in our study (data not shown) and most published pan-TDP-43 antibodies. Our previous findings that TDP-43 could be extracted biochemically with low-stringency buffers from cultured cells [27], but was only extractable with high-stringency detergent-containing buffer from CNS tissue [1], supports a potential structural difference between nuclear TDP-43 in CNS and cultured cells. Thus, the slight immunoreactivity of MAb 138 with non-pathological TDP-43 in non-neuronal cell culture is difficult to interpret precisely at this time, but we will continue to characterize the basis for its preferential recognition of pathological TDP-43 in a large comprehensive neuropathological survey and further neuronal culture experiments.

Protein misfolding and prion-like spread of neurodegenerative disease proteins has emerged in the forefront of research for drug-development in FTLD-TDP and ALS [28,29]. Although TDP-43 transgenic mouse models with cytoplasmic TDP-43 accumulations that recapitulate clinical and pathological phenotypes of the human disease remain to be developed, novel MAbs such as those reported here will be critical to evaluate the sequential deposition of pathological TDP-43 for pre-clinical evaluations of novel therapeutics focused on TDP-43 aggregation as well as for use as potential immune therapies. Finally, there is an urgent need for FTLD-TDP/ALS-specific biomarkers [30], and ELISAs that are able to reliably measure CSF and/or plasma levels of TDP-43 will be useful not only for diagnosis, but also for monitoring responses to disease modifying therapies for FTLD-TDP, ALS and other TDP-43 proteinopathies [31]. Hence, we expect that the panel of novel MAbs described here will be informative tools for future patient oriented and experimental studies of TDP-43 proteinopathies.

## Competing interests

The authors declare that there are no competing interests to disclose.

## Authors’ contributions

LKK, DJI, AKW, VML, and JQT designed the study and wrote the manuscript. DMR carried out the hybridoma fusion and subcloning as well as the ICC experiments. LKK, AKW, and YX performed the MAb characterizations and evaluations. DJI evaluated the human neuropathology. All authors read and approved the final manuscript.

## Supplementary Material

Additional file 1: Figure S1 Representative ELISA results for a newly developed MAb, **Figure S2.** Serial Dilutions of MAb 138 show minimal nuclear reactivity and **Table S1.** Summary of patient demographics. Click here for file
