# Supplementary material for: Novel monoclonal antibodies to normal and pathologically altered human TDP-43 proteins
Source: Acta Neuropathol Commun. 2014 Mar 31;2:33. doi: 10.1186/2051-5960-2-33 (PMC4023626; doi:10.1186/2051-5960-2-33)
Supplement: Additional file 1: Figure S1 — Representative ELISA results for a newly developed MAb, Figure S2. Serial Dilutions of MAb 138 show minimal nuclear reactivity and Table S1. Summary of patient demographics. [file 2051-5960-2-33-S1.pdf]

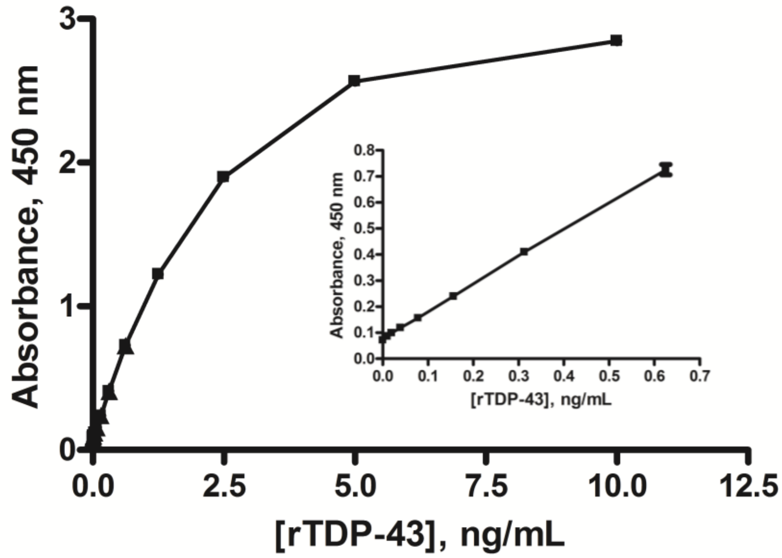

Fig. 1S Representative ELISA results for a newly developed MAb. Representative data from a sandwich ELISA using MAb 205 as capture and a rabbit polyclonal antibody to the C-terminus of TDP-43 using human rTDP-43 is shown. The lower limit of detection of human rTDP-43 for this ELISA is <25 pg/mL and the linear range for this ELISA is shown in the insert

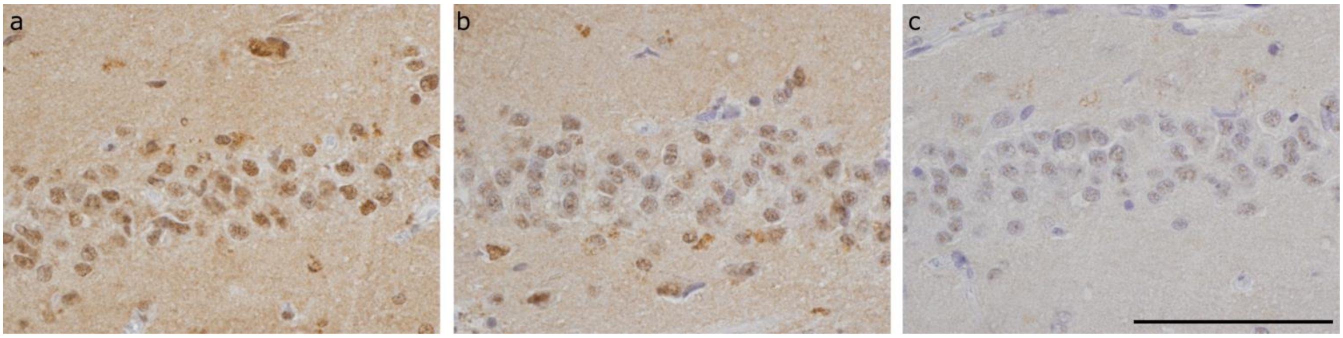

Fig. 2S Serial Dilutions of MAb 138 show minimal nuclear reactivity. Shown here are IHC results of serial dilutions of purified MAb 138 IgG from 0.3 μg/ml (a), 0.1 μg/ml (b) and 0.03 μg/ml (c) in normal control human dentate gyrus. Note that when this IgG is used at a concentration of 0.03 μg/ml, both the background neuropil staining and nuclear staining are eliminated while at this concentration MAb 138 recognizes pathological TDP-43 inclusions as shown in Fig. 4. Scale bar= 100 μm

Table-S1. Summary of patient demographics

Neuropathological diagnosis	Age at Death, years	Sex	PMI, hours	Brain-weight, grams	Fixative
ALS	55	F	6	1480	NBF
FTLD-TDP type A	49	F	6	591	NBF
FTLD-TDP type B*	73	F	11	1033	NBF
FTLD-TDP type C	76	M	NA	1200	NBF
FTLD-TDP type D	70	F	6	1022	ETOH
Normal Control	68	M	21	1330	NBF
Alzheimer Disease Control	85	M	24	1440	ETOH

Abbreviations: PMI= post-mortem interval, NA= not available, ETOH= 70% ethanol with 150 mmol sodium chloride, NBF= 10% neural-buffered formalin.

*This case had secondary diagnoses of Transitional Stage Lewy body disease and hippocampal sclerosis.
